# Subsidence of Uncemented Short Stems in Reverse Shoulder Arthroplasty—A Multicenter Study

**DOI:** 10.3390/jcm9103362

**Published:** 2020-10-20

**Authors:** Anna-K. Tross, Alexandre Lädermann, Thomas Wittmann, Marc Schnetzke, Philip-C. Nolte, Philippe Collin, Patric Raiss

**Affiliations:** 1 Clinic for Orthopedics and Trauma Surgery, Heidelberg University Hospital, Schlierbacher Landstraße 200a, 69118 Heidelberg, Germany; anna-katharina.tross@med.uni-heidelberg.de; 2 Division of Orthopaedics and Trauma Surgery, La Tour Hospital Meyrin, Avenue Jacob-Daniel Maillard 31217 Meyrin, Switzerland; alexandre.laedermann@gmail.com; 3 Faculty of Medicine, University of Geneva, Rue Michel Servet 1, 1211 Geneva, Switzerland; 4 Division of Orthopaedics and Trauma Surgery, Department of Surgery, Geneva University Hospitals, Rue Gabrielle-Perret-Gentil 4, 1205 Geneva, Switzerland; 5 OCM (Orthopädische Chirurgie München), Steinerstrasse 6, 81369 Munich, Germany; th-wittmann@gmx.de; 6 German Joint Centre, ATOS Clinic Heidelberg Bismarckstraße 9, 69115 Heidelberg, Germany; marcschnetzke@gmx.de; 7 BG Trauma Center Ludwigshafen at the University of Heidelberg, Clinic for Trauma and Orthopaedic Surgery, Ludwig-Guttmann-Strasse 13, 67071 Ludwigshafen on the Rhine, Germany; pnolte@sprivail.org; 8 Centre Hospitalier Privé Saint-Grégoire (Vivalto Santé), 6 Boulevard de la Boutière, 35768 Saint-Grégoire, France; docphcollin@gmail.com

**Keywords:** shoulder, reverse arthroplasty, uncemented, short-stem, prostheses, subsidence, humeral loosening, filling ratio

## Abstract

Background: The radiological phenomenon of subsidence following the implantation of uncemented short-stem reverse prostheses (USSP) has not yet been described. The purpose of this study was to describe the rate and potential risk factors for subsidence. We hypothesized that subsidence may be a frequent finding and that a subsidence of >5 mm (mm) is associated with an inferior clinical outcome. Methods: A total of 139 patients with an average age of 73 ± 9 years were included. The clinical and radiological outcome was evaluated at a minimum follow-up (FU) of 12 months. Results: No humeral component loosening was present at a mean FU of 18 (range, 12–51) months. Mean Constant Score (CS) and Subjective Shoulder Value (SSV) improved significantly from 34.3 ± 18.0 points and 37.0 ± 19.5% preoperatively to 72.2 ± 13.4 points and 80.3 ± 16.5% at final FU (*p* < 0.001). The average subsidence of the USSP was 1.4 ± 3.7 mm. Subsidence of >5 mm was present in 15 patients (11%). No association between a subsidence >5 mm and CS or SSV was found (*p* = 0.456, *p* = 0.527). However, a subsidence of >5 mm resulted in lower strength at final FU (*p* = 0.022). Complications occurred in six cases (4.2%), and the revision rate was 3.5% (five cases). Conclusions: Although subsidence of USSP is a frequent radiographic finding it is not associated with loosening of the component or a decrease in the clinical outcome at short term FU. Level of evidence: Level 4, retrospective study.

## 1. Introduction

Since Charles Neer [1] introduced the first model of an “articular replacement prosthesis” for fractures of the humeral neck with dislocations of the head fragment in 1953, shoulder arthroplasty has become a well-established surgical option for the treatment of degenerative diseases of the glenohumeral joint [2,3]. In the following years, anatomical cemented or uncemented standard length (around 100 mm; mm) stem prostheses were frequently used and became the gold standard [4,5,6,7,8,9].

Long-term survival of components as well as bone preserving surgical techniques seem to be crucial since demographic changes lead to an increased number of individuals aged 60 or older, potentially necessitating joint replacement [10].

Humeral component long-term survival may be affected by the mode of fixation (press-fit versus cemented) [11]. Uncemented “press-fitted” stems offer the advantage of reduced operating time, less risk of thromboembolism, and the avoidance of cement at the time of revision surgery [12,13,14]. However, as a long-term disadvantage, press-fit fixation of the stem can lead to metaphyseal bone resorption caused by proximal micromotion or “stress shielding” following Wolff’s law as previously described [12,15,16,17,18]. As a result, the bone becomes either thinner (external remodeling) or more porous (internal remodeling) [15]. Loosening of the humeral component is defined by implant migration or a radiolucent line of two or more millimeters around the entire stem seen on X-rays and has been described for standard stems in some studies [12,19,20,21].

Uncemented short-stem prostheses (USSP) have been introduced as a bone preserving solution for the replacement of the proximal humerus. Ideally, a USSP is convertible from an anatomic to a reverse replacement or vice versa and should be easy to revise without severe bony destruction [5,22,23].

In the past, different radiological changes after the implantation of USSP for total shoulder arthroplasty (TSA)s and reverse shoulder arthroplasty (RSA)s were described. Radiologic bone adaptions seem to be related to higher filling ratios and cortical contact of the stem [24,25,26,27]. Additionally, subsidence was described as one of the criteria for component loosening in standard stems [8,20]. The purpose of this study was to describe the rate and potential risk factors for subsidence of reverse USSPs. We hypothesized that subsidence may be a frequent finding in reverse USSPs and that a subsidence of greater than 5 mm is associated with an inferior clinical outcome.

## 2. Experimental Section

A retrospective comparative study was conducted on prospectively collected data on RSAs performed at three different specialized shoulder centers between January 2013 and December 2018. Institutional review board approval was obtained prior to the start of the study: Commission cantonale d’éthique de la recherche scientifique (CCER) of Geneva (blinded for review). Each of the 3 surgeons performed >100 arthroplasty cases per year and all surgeons were fellowship trained by the same surgeon with a special focus on short stem arthroplasty. Inclusion criteria were (1) minimum follow-up (FU) of 12 months, (2) complete radiographic data immediately postoperatively and at final follow-up, (3) no previous arthroplasty surgery and (4) primary treatment with the same type of short stem RSA (Ascend^TM^ Flex, Memphis, TN, USA). Cases of suboptimal rotation in the anteroposterior x-ray view were excluded. A strict anteroposterior view was defined by the visualization of the morse taper as described by Goetzmann et al. [28]. A reverse USSP was implanted in 468 cases. Among these, the criteria of complete radiographic data or of a minimum follow-up of 12 months were not fulfilled in 107 reverse USSPs. Due to suboptimal rotation in the anteroposterior x-ray view 218 reverse USSPs had to be excluded. 

**Figure 1:**
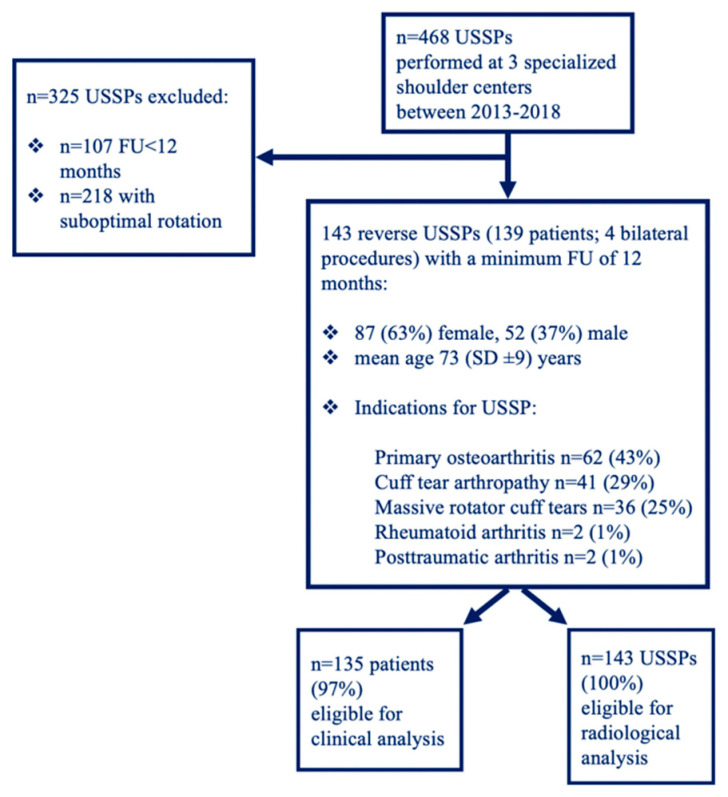
Flow chart of exclusion and inclusion criteria of the study cohort. USSP: Uncemented Short Stem Prostheses, FU: Follow-Up.

The overall cohort included in this study consisted of 143 reverse USSPs (139 patients; four bilateral procedures) with a mean radiological follow-up of 18 (standard deviation (SD) ± 8; range, 12–51) months and a mean clinical follow-up of 17 (SD ± 7; range 12–51) (Figure 1). The most common indication for reverse USSP was primary osteoarthritis (43%) and the second most common indication was cuff tear arthropathy (29%). A total of 87 patients were female (63%), and 52 were male (37%). Patients had a mean age of 73 (SD ± 9) years, the right shoulder was operated on in 104 (73%) cases and the left in 39 (27%) cases. Bony increased offset (Bio)-RSA was used in a total of 92 cases (64%) according to the method described by Boileau et al. [29]. Demographics and characteristics are shown in Table 1.

### 2.1. Implant

The reverse USSP used in this study was described previously [25]. It is made of titanium with a proximal porous coating on the metaphyseal section for bone ingrowth and ranges in length from 66 to 94 mm based on diameter. A stem with an inclination of 132.5 degrees was used with a humeral tray available in 2 different offsets (1.5 mm and 3.5 mm). Connected to the tray is a polyethylene insert with 12.5 degrees of inclination, resulting in a humeral inclination angle of 145 degrees.

### 2.2. Surgical Technique

The exact surgical technique was described before [25]. In all cases, a classic deltopectoral approach was used. After tenotomy of the subscapularis the muscle was released. Osteophytes were removed and the anatomical neck was identified. Then, using a free-hand technique, the humeral head was resected. Exposition of the glenoid was achieved using four retractors. Reaming was performed over the pin guide with the goal to preserve as much bone layer as possible in each case. The baseplate was fixed uncemented and the glenosphere was placed. Attention was then paid to the humeral canal which was opened and sized with canal finders. With compactors, that have the same shape as the humeral implant, the canal was successively broached in preparation of the cancellous bone for the final implant. The definitive implant was then placed. To avoid lengthening of the arm, a humeral tray was positioned slightly below the tip of the greater tuberosity and the liner was placed. In the final step the joint was reduced, the arm was examined for stability and appropriate tension. When possible, a tendon-to-tendon repair was performed with nonabsorbable sutures [30]. The wound was closed in a layered fashion.

### 2.3. Clinical Analysis

The Constant Score (CS) with its subcategories and the Simple Shoulder Value (SSV) as well as range of motion for shoulder flexion and external rotation were measured for 135 reverse USSPs before surgery and at the most recent follow-up. Two reverse USSPs of the study cohort could not be included because of incomplete clinical follow-up. Demographics, diagnoses, postoperative complication and revision rates were taken from the patients’ medical records. Clinical results of patients who developed a postoperative complication were reported separately.

### 2.4. Radiographic Analysis

**Figure 2:**
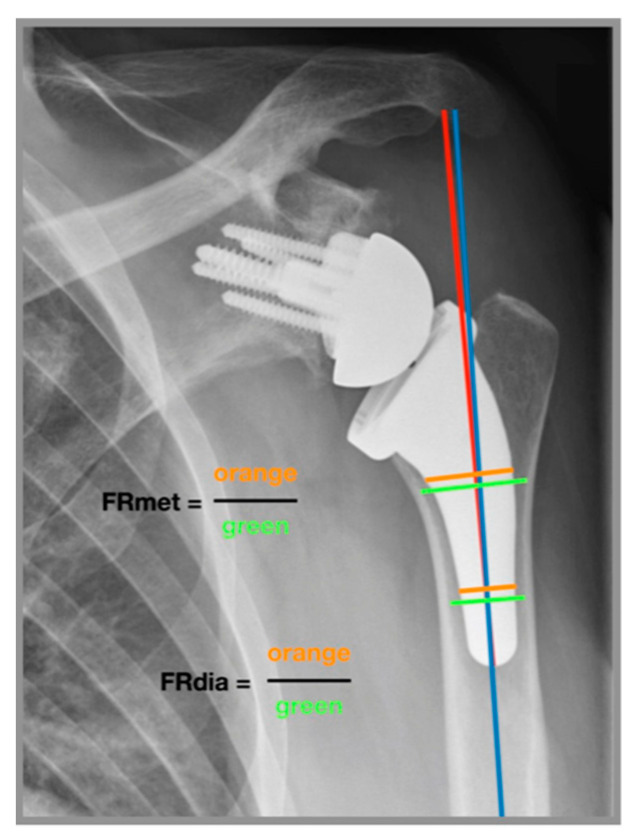
Measurement of filling ratio and alpha angle [27]. Anteroposterior radiograph of a left shoulder. The alpha angle (*α*) was measured between the shaft axis (blue line) and the stem axis (red line). Filling ration at the level of the metaphysis (FRmet) is a line perpendicular to the shaft axis, intersecting at the distal–medial border of the humeral platform. Filling ratio at the level of the diaphysis (FRdia) is a line perpendicular to the shaft axis, intersecting at the distal third of the prosthesis. The filling ratio is the quotient of the orange and green lines at the metaphysis (FRmet) and at the diaphysis (FRdia). Distances were calibrated based on the size of the glenosphere.

**Figure 3:**
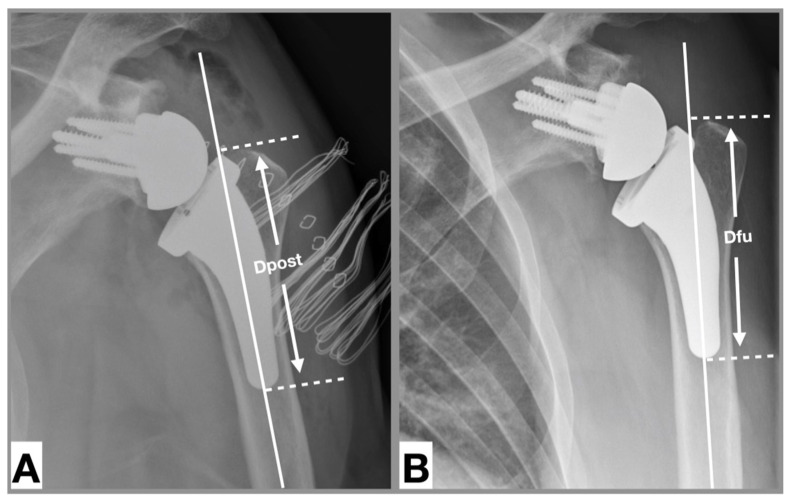
Measurement of subsidence [33]. Anteroposterior radiograph of a left shoulder. (**A**). Postoperative, (**B**). At 12 months follow-up. Shaft axis (solid white line), tip of tuberosity (white dotted line perpendicular to shaft axis), distal end of trabecular metal (white dotted line perpendicular to shaft axis). Subsidence: Distance between white dotted line at follow-up minus distance between white dotted line postoperative. Distances were calibrated based on the size of the glenosphere.

Two independent examiners performed radiographic measurements of 137 reverse USSPs on comparable anteroposterior radiographs immediately postoperatively and at final follow-up. Stem inclination (neutral/valgus/varus) was measured in degrees and the filling ratio of the humeral shaft was measured at the level of the metaphysis and diaphysis in millimeters according to the method introduced by Schnetzke et al. (Figure 2) [27]. If the angle of the stem relative to the humeral canal was ± 5 degrees, the inclination was considered as neutral. If the angle was >5 degrees the inclination was considered as valgus and if the angle was <5 degrees the inclination was considered as varus [31]. Cortical thickness was measured according to the method of Mather et al. [32]. Subsidence was determined by comparing the distance between the most cephalic aspect of the greater tuberosity with the distal border of the stem according to the method introduced by Bogle et al. (Figure 3) [33] and was empirically defined as an inferior migration of the shaft greater than 5 mm. Radiological results of patients who developed a postoperative complication were reported separately.

### 2.5. Statistics

Means and Standard Deviation (SD) were calculated for continuous variables. Differences between pre-and postoperative data were calculated using the Wilcoxon Test for non-normally distributed data. Differences between postoperative means and between two groups were calculated using Student’s t-Test for normally distributed and the Mann–Whitney-U Test for non-normally distributed data. The χ^2^ test was used for the analysis of contingency tables. A Pearson’s Chi-Squared Test was performed to determine statistical differences between subgroups. The level of significance was set at *p* < 0.05. Calculation of the interobserver agreement was performed with the Cohen κ, and agreement strength was deduced in regard to the recommendations of Landis and Koch.

## 3. Results

### 3.1. Clinical Results

At final follow-up, mean CS and mean SSV improved significantly from 34.3 (SD ± 18.0) points and 37.0 (SD ± 19.5)% preoperatively to 72.2 (SD ± 13.4) points and 80.3 (SD ± 16.5) % at final follow-up (*p* < 0.001 and *p* < 0.001). All subcategories of the CS (activity, mobility, strength and pain) demonstrated statistically significant higher scores at final follow-up (*p* < 0.001). External rotation and flexion improved from 25.8 (SD ± 25.2) degrees and 108.4 (SD ± 49.3) degrees to 36.7 (SD ± 21.6) degrees and 144.0 (SD ± 23.4) degrees (*p* < 0.001 and *p* < 0.001) (Clinical outcomes Table 2). CS, SSV, flexion and external rotation were not associated with age (*p* = 0.336, *p* = 0.250, *p* = 0.265, *p* = 0.511). However, female sex did have a positive impact on the postoperative CS (*p* = 0.007) while SSV, flexion and external rotation did not (*p* = 0.655, *p* = 0.089, *p* = 0.136).

### 3.2. Complications

During the study period, six complications (4.2%) occurred. One patient developed a postoperative hematoma, which had to be drained surgically one week postoperative. Two patients suffered from a periprosthetic fracture (three and five months postoperative). Of those, one patient had to undergo open reduction and internal fixation with a plate osteosynthesis while the other patient was treated non-operatively in a sling. In one case of instability, revision with removal of the short stem and replacement by a long stem became necessary one month after the initial operation. One patient suffered from a late-onset (16 months postoperative) local infection and had to be treated with a spacer. One patient was diagnosed with a suspicious bone lesion underneath the stem one month after the initial operation and had to be revised to a cemented long stem (complications Table 3).

### 3.3. Radiological Outcome

Mean subsidence at final follow-up was 1.4 (SD ± 3.7) mm (radiological outcome Table 4). A subsidence of greater than 5 mm was demonstrated in 15 cases (11%). Subsidence was not influenced by patients’ age (*p* = 0.164), sex (*p* = 0.463) or metaphyseal or diaphyseal filling ratio (*p* = 0.484, *p* = 0.603). Moreover, metaphyseal or diaphyseal cortical thickness were not associated with the risk of subsidence (*p* = 0.480, *p* = 0.476). With 13 of 15 cases of subsidence of more than 5 mm in the osteoarthritis group, a positive correlation between the factor indication for USSP and subsidence was found (*p* = 0.011). The use of Bio-RSA did not influence the presence of subsidence >5 mm (*p* = 0.884). No correlation between the postoperative shaft inclination angle or the shaft inclination angle at final follow-up and the risk of subsidence was present (*p* = 0.480, *p* = 0.348). No association between CS or SSV and the risk of subsidence could be demonstrated (*p* = 0.456, *p* = 0.527); however, patients with a subsidence >5 mm were found to reach a significantly lower strength level (mean 8.3; SD ± 6.4) than patients with a subsidence <5 mm (mean 11.5; SD ± 5.8) (*p* = 0.022). Means for CS were lower for patients with a subsidence >5 mm (mean 71.2; SD ± 13.6) than for patients with a subsidence <5 mm (mean 72.3; SD ± 13.4) (*p* = 0.456). Means for the CS subcategories mobility and pain were lower for patients with a subsidence >5 mm (mean 29.7; SD ± 8.0 and mean 19.2; SD ± 19.7) than for patients with a subsidence <5 mm (mean 31.1; SD ± 6.1 and mean 29.9; SD ± 26.8) (*p* = 0.658, *p* = 0.189). Furthermore, patients with a subsidence >5 mm achieved less flexion and external rotation (mean 138.3; SD ± 24.6 and mean 36.7; SD ± 16.8) than patients with a subsidence > 5 mm (mean 144.7; SD ± 23.3 and mean 36.8; SD ± 22.3) (*p* = 0.231, *p* = 0.960).

The mean filling ratio of the metaphysis was 0.61 (SD ± 0.12), the mean filling ration of the diaphysis was 0.70 (SD ± 0.11). Male patients had a significantly lower metaphyseal and diaphyseal filling ratio (mean 0.59; SD ± 0.11 and mean 0.64; SD ± 0.09) compared to women (mean 0.68; SD ± 0.10 and mean 0.76; SD ± 0.10) (*p* = 0.029 and *p* < 0.001). The mean cortical width in the metaphysis was 3.6 (SD ± 1.5) mm and in the diaphysis 5.4 (SD ± 1.3) mm. The mean postoperative inclination of the stem was 3.9 (SD ± 3.1) degrees valgus, whereas the mean inclination of the shaft at follow-up was 5.1 (SD ± 9.6) degrees valgus. At final follow-up a total of 97 patients (71%) demonstrated a neutral inclination angle of the stem, whereas valgus position was observed in 21% (*n* = 29), and varus position in 8% (*n* = 11) of cases. The interrater reliability between two independent observers was considered as almost perfect (k = 0.825). For detailed means, standard deviation and risk factors for subsidence see Table 5.

The impact of the follow-up range (12–51 months) in this study was further analyzed. Only three patients of the study cohort had a follow-up over 36 months. It was found that subsidence was not influenced by the wide follow-up range (*p* = 0.539).

## 4. Discussion

The purpose of this study was to evaluate the frequency of subsidence of the humeral stem and potential risk factors after RSA with a USSP. The first and most important finding was that a subsidence of greater than 5 mm occurred frequently and was present in 15 patients (11%) of this study cohort. The average subsidence of the shaft was 1.4 (SD ± 3.7) mm after a mean of 18 (SD ± 8) months. With the measurement technique according to Bogle et al. [33], it was possible to describe the exact extent of subsidence as demonstrated in our study, and the standardized application will help to compare our results to those of future investigations. A finding of practical note is that true anteroposterior x-ray views are required for the exact measurement of subsidence.

The phenomenon of subsidence was discussed frequently in the past. In 2000 Sperling et al. [8] defined humeral components as “at risk” when there was radiographic evidence of subsidence, tilt, or 2 mm radiolucent lines around the implant following primary (regular long stem) implantation of TSAs. Between 2015 and 2018, several studies evaluated radiographic results after TSA with an uncemented short stem prosthesis [26,27,34,35,36]. Only in two studies, small numbers of subsidence were found, but the extent of the inferior migration was not expressed numerically [34,35]. In 2016, Morwood et al. [35] analyzed the effect of proximal porous coating on a mini-stem humeral component in 68 shoulders with glenohumeral osteoarthritis, proximal humeral fracture sequelae, avascular necrosis of the humeral head, or instability arthropathy. After a two-year follow-up of 34 coated stems, no stem loosened, but one showed subsidence and seven developed radiolucency. Of 34 uncoated stems, one showed aseptic loosening and required revision surgery after 26 months, seven were judged at risk of loosening (two because of subsidence), and 15 developed radiolucency. There was no difference in final range of motion or outcome scores between the two intervention groups. In 2017, Denard et al. [34] compared the outcomes and radiographic humeral adaptions following placement of a traditional-length humeral component in 58 shoulders or short-stem humeral component (Univers II; Arthrex, Inc., Naples, FL, USA) in 56 shoulders after TSA. There was no difference in functional outcomes after two years between the intervention groups. One stem in the traditional-length group and three stems in the short-stem group showed subsidence or shift. None of the stems were revised and all patients were satisfied with their results. Statistical analysis showed that there was no difference in loosening or shift between the two groups. The length of follow-up for the previously described studies can be considered as short, and future long-term studies are needed to demonstrate if and how subsidence influences loosening rates and the clinical outcome. 

Five studies evaluated the phenomenon of subsidence between 2014 and 2019 for reverse USSPs [22,27,37,38,39]. Atoun et al. [22] examined the radiographic outcome including subsidence of a reverse USSP (Verso; Biomet, Swindon, UK) in 31 patients after a mean follow-up of 36 months. No humeral loosening or subsidence was observed. Schnetzke et al. [27] reported on the radiological outcome including subsidence of 24 patients treated with a reverse USSP (Ascend^TM^ Flex Shoulder System; Fa. Wright, Memphis, TN, USA). At 25 months follow-up none of the stems showed evidence of subsidence or loosening. Merolla et al. [38] evaluated the radiological outcome including subsidence, after implanting a reverse USSP (Tornier Aequalis Ascend^TM^ Flex Shoulder System, Memphis, TN, USA) in 38 shoulders. No subsidence was present at a minimum follow-up of 24 months. Ascione et al. [37] assessed the radiological outcome including subsidence after the implantation of a reverse USSP (Tornier Aequalis Ascend^TM^ Flex Shoulder System) in 100 patients. No case of subsidence occurred at final follow-up of a minimum of two years. Peduzzi et al. [39] evaluated the radiological outcome including subsidence of 81 shoulders after surgical treatment with a reverse USSP (Tornier Ascend^TM^ Flex Shoulder System, Memphis, TN, USA) at a minimum follow-up of 24 months, but no case of subsidence was present. Again, all studies evaluated the radiographic outcomes only at short-term follow-up and neither subsidence nor aseptic loosening was described as a potential “risk” within this timeframe. A possible explanation for our frequent findings may be the exact measurement method we used; however, the aforementioned studies did not define their method of measurement. Interestingly, a subsidence of more than 5 mm was found to be associated with osteoarthritis. Studies in which subsidence of short stems was described before did not report about an association with osteoarthritis [34,35].

In line with these short-term results after RSA, no implant loosening was found at a minimum follow-up of 12 months in our study. One could argue that a subsidence of greater than 5 mm can be judged as loosening, however, no patient had to be revised due to stem loosening until final follow-up. A continuous and careful observation of these patients is necessary in order to draw conclusions as to if the observed subsidence in the present trial has an influence on long-term survival of the humeral component.

We hypothesized that subsidence may be a frequent finding in USSP and that a subsidence of greater than 5 mm is associated with an inferior clinical outcome. As the second most important finding this study demonstrated that, although subsidence of USSP is a frequent radiographic finding, it is not associated with inferior shoulder function (CS, SSV and range of motion) at short term follow-up. However, patients with a subsidence of greater than 5 mm achieved a statistically significant lower strength level in the subcategory analysis of the CS. Lower scores for mobility and pain without statistical significance were present in the group of patients with a subsidence greater than 5 mm. Patients with a subsidence greater than 5 mm reached smaller degrees in range of motion without statistical significance. A potential association between shoulder function and subsidence has not been evaluated in studies on reverse USSP before. Our results implicate that a subsidence of greater than 5 mm may be related to inferior clinical outcome scores in the longer follow-up so we recommend constant clinical controls for affected patients.

As a third finding our statistical analysis revealed that men have a significantly lower metaphyseal and diaphyseal filling ratio compared to women. We assume that in men, because of their stronger bone structure, the stem is anchored in the cancellous bone, rather than on the inner cortical bone, thus resulting in a lower filling-ratio. This assumption is based on the average age of our patient cohort (mean 73; SD ± 9 years) and a probably better bone structure of men compared to women who are often affected by postmenopausal osteoporosis [40,41]. In a reverse conclusion, female patients were shown to have a higher metaphyseal and diaphyseal filling ratio than men. Mather et al. [32] previously showed that a cortical thickness of less than 6 mm is a potential threshold value for predicting osteoporosis. Raiss et al. [25] demonstrated that female sex, cortical thickness smaller than 6 mm and a close cortical contact was associated with high bone adaptions after TSA and RSA with a USSP. All this leads to the speculation that women possess a higher risk for high bone adaptions. The upcoming question is if there is a gender determined consideration for future implantations. The association between higher filling ratios and high stress shielding related bone adaptation rates has been shown previously in TSA and RSA [23,25,27,42]. It is therefore recommended to avoid oversizing the stem [25] and to avoid a close to press-fit fixation of the short stem to minimize the occurrence of humeral bone remodeling [27]. Both phenomena, subsidence and high bone adaptions, are expected to be risk factors for long-term stem loosening [8].

**Figure 4:**
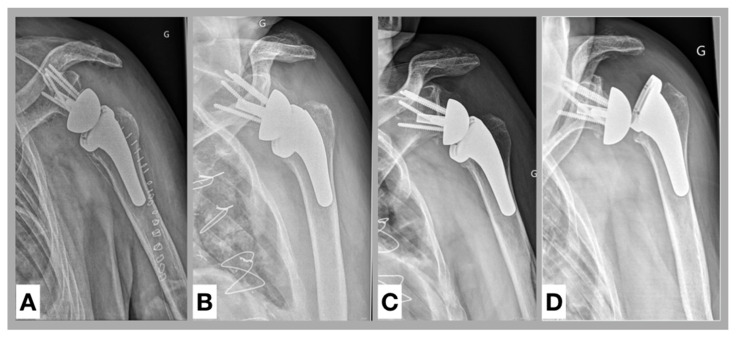
Complication following subsidence. Anteroposterior radiographs of a left shoulder. (**A**). Postoperative, (**B**). 3 months follow-up, (**C**). 6 months follow-up, (**D**). 9 months follow-up: Due to subsidence a dissociation between the tray and the stem (Morse taper) occurred.

In the past, subsidence was described as a phenomenon rather related to glenoid loosening [43]. Boileau et al. [44] claimed that fixation of a RSA may be more problematic on the humeral side than on the glenoid side, and some of the potential reasons for these complications such as humeral subsidence or loosening may be related to the fact that the humeral stem is round and offers very little resistance to rotational torque. A possible complication following subsidence is a dissociation between the tray and the stem and was found during our patient recruitment (Figure 4). Although this specific case had to be excluded due to suboptimal rotation in the anteroposterior X-ray view, surgeons should be alert to this potential complication.

It was 2014 when Giuseffi et al. [23] stated that the outcome of short cementless humeral stems in RSA, which may be prone to malalignment or poor fixation, is not known. Thus, the use of a short stem in RSA is theoretically worrying, since the semi-constrained nature of RSA could lead to increased torsional stress, micromotion, and failure. Six years later we consider data, that show good short-term data but also worrying “risk signs” for a potential long-term stem loosening. The mid- and long-term results of short stem RSAs need to be assessed in future studies.

### Limitations

This study has limitations. First, the time of observation (within 12 months) is short and the range of follow-up is wide (12–51 months). Longer follow-up is crucial to evaluate how radiographic findings change over time and if they result in loosening. Statistical analysis revealed that only three patients of the study cohort had a follow-up of more than 36 months and no impact on the presence of a subsidence of more than 5 mm was found (*p* = 0.539).

Second, only one type of short stem was analyzed, and we did not compare our results to a control group.

## 5. Conclusions

Although subsidence of USSP is a frequent radiographic finding, it is not associated with loosening of the component or a decrease in the clinical outcome at short term follow-up. However, the relevance of subsidence for future loosening of the stem remains unclear.

## Figures and Tables

**Table 1:** Demographics and characteristics of the study cohort.

	Demographics and Characteristics
Shoulders, *n*	143
Age (Years)	73 (SD ± 9)
Gender, Female/Male *n* (%)	87 (63)/52 (37)
Primary Osteoarthritis, *n* (%)	62 (43)
Cuff Tear Arthropathy, *n* (%)	41 (29)
Massive Rotator Cuff Tears, *n* (%)	36 (25)
Rheumatoid Arthritis, *n* (%)	2 (1)
Posttraumatic Arthritis, *n* (%)	2 (1)
Uncemented RSA, *n* (%)	143 (100)
Bio-RSA, *n* (%)	92 (64)
Radiological Follow-Up (Months)	18 (SD ± 8)
Clinical Follow-Up (Months)	17 (SD ± 7)

Demographics and characteristics are reported in means and standard deviation (Bio-RSA = bony increased offset reversed shoulder arthroplasty, RSA = reverse shoulder arthroplasty, SD = standard deviation).

**Table 2:** Clinical outcomes.

	Preoperative	At Follow-Up	*p*-Value
SSV (%)	37.0 (SD ± 19.5)	80.3 (SD ± 16.5	*p* < 0.001 *
CS (Points)	34.3 (SD ± 18.0)	72.2 (SD ± 13.4)	*p* < 0.001 *
Activity (Points)	8.6 (SD ± 4.1)	16.6 (SD ± 3.4)	*p* < 0.001 *
Mobility (Points)	19.0 (SD ± 10.1)	30.9 (SD ± 6.4)	*p* < 0.001 *
Strength (Points)	3.3 (SD ± 4.4)	11.1 (SD ± 5.9)	*p* < 0.001 *
Pain (Points)	7.3 (SD ± 4.6)	28.6 (SD ± 26.2)	*p* < 0.001 *
Flexion (Degree)	108.4 (SD ± 49.3)	144.0 (SD ± 23.4)	*p* < 0.001 *
External Rotation (Degree)	25.8(SD ± 25.2)	36.7 (SD ± 21.6)	*p* < 0.001 *

Clinical outcome preoperative and at final follow-up is reported in means and standard deviation for 135 patients. The statistical level of significance was calculated using the Wilcoxon Test (*). SSV: Simple Shoulder Value, CS: Constant Score.

**Table 3:** Complications and revisions among the study cohort. Complications after 143 reverse USSPs.

Complications	Frequency	Time until Complication	Revision Surgery
Postoperative Hematoma	1	7 Days	Removal of Hematoma
Periprosthetic Fracture	2	3 Months 5 Months	Plate Osteosynthesis Non-Operative
Dislocation	1	1 Month	Short Stem Removal, Long Stem Implantation
Infection	1	16 Months	Short Stem Removal, Spacer Implantation
Neoplasia Under the Stem	1	1 Month	Short Stem Removal, Long Stem Implantation

**Table 4:** Radiological outcome.

	Radiological Outcome
Subsidence (mm)	1.4 (SD ± 3.7)
Subsidence >15 mm, *n* (%)	15 (11)
Metaphyseal FR	0.61 (SD ± 0.12)
Diaphyseal FR	0.70 (SD ± 0.11)
Cortical Width Metaphyseal (mm) Postoperative	3.6 (SD ± 1.5)
Cortical Width Diaphyseal (mm) Postoperative	5.4 (SD ± 1.3)
Shaft Inclination Postoperative	99 Neutral (72%), 29 Valgus (21%), 9 Varus (7%)
Shaft Inclination at Final FU	97 Neutral (71%), 29 Valgus (21%), 11 Varus (8%)

FR: Filling Ratio, FU: Follow-Up.

**Table 5:** Risk factors for subsidence.

	Subsidence < 5 mm	Subsidence > 5 mm	*p*-Value
Age (years)	72.71 (SD ± 8.86)	76.8 (SD ± 7.03)	0.164 *
SSV at FU (%)	80.24 (SD ± 17.32)	80.71(SD ± 7.87)	0.527 *
CS at FU (Points)	72.31 (SD ± 13.38)	71.19 (SD ± 13.57)	0.456 *
Activity at FU (Points)	16.51 (SD ± 3.54)	17.29 (SD ± 2.33)	0.634 *
Mobility at FU (Points)	31.06 (SD ± 6.14)	29.67(SD ± 8.04)	0.658 *
Strength at FU (Points)	11.53 (SD ± 5.77)	8.33 (SD ± 6.39)	0.022 *
Pain at FU (Points)	29.85 (SD ± 26.75)	19.19 (SD ± 19.66)	0.189 *
Flexion at FU (Degree)	144.72 (SD ± 23.3)	138.33 (SD ± 24.62)	0.231 *
Extension at FU (Degree)	36.81 (SD ± 22.28)	36.67 (SD ± 16.76)	0.960 *
Diaphyseal FR	0.69 (SD ± 0.11)	0.71 (SD ± 0.09)	0.603 *
Metaphyseal FR	0.61 (SD ± 0.12)	0.64 (SD ± 0.13)	0.484 **
Metaphyseal Cortical Width (mm)	3.61 (SD ± 1.43)	3.40 (SD ± 1.76)	0.480 *
Diaphyseal Cortical Width (mm)	5.47 (SD ± 1.27)	5.33 (SD ± 1.45)	0.476 *
Inclination FU (Degree)	5.26 (SD ± 10.03)	4.17 (SD ± 4.89)	0.348 *

Risk factors for subsidence are reported in means and standard deviation. The statistical level of significance was calculated using the Mann–Whitney-U test (*) and the Students-t-test (**). CS: Constant score, FR: Filling Ratio. FU: Follow-Up, SSV: Simple Shoulder Value.
